# Outcomes of Severe Sepsis and Septic Shock Patients on Chronic Antiplatelet Treatment: A Historical Cohort Study

**DOI:** 10.1155/2013/782573

**Published:** 2013-02-20

**Authors:** Juan C. Valerio-Rojas, Insara J. Jaffer, Daryl J. Kor, Ognjen Gajic, Rodrigo Cartin-Ceba

**Affiliations:** ^1^Multidisciplinary Epidemiology and Translational Research in Intensive Care (METRIC), Mayo Clinic, Rochester, MN 55905, USA; ^2^Department of Anesthesiology, Mayo Clinic, Rochester, MN 55905, USA; ^3^Division of Pulmonary and Critical Care Medicine, Mayo Clinic, 200 First St SW, Rochester, MN 55905, USA

## Abstract

*Background*. Sepsis is characterized by dysfunctional activation of platelets, and antiplatelet therapy could improve the outcomes of septic patients. *Methods*. We performed a retrospective cohort study of severe sepsis or septic shock adult patients. Outcomes of patients on antiplatelet therapy were compared to those that were not taking antiplatelet therapy by univariate analysis followed by a propensity score analysis based on the probability of receiving antiplatelet therapy. *Results*. Of 651 patients included in the study 272 (42.8%) were on antiplatelet therapy before the development of severe sepsis or septic shock. After adjusting for important confounding variables antiplatelet therapy was not associated with a decreased risk of hospital mortality (odds ratio 0.73, 95% confidence interval 0.46–1.16). Antiplatelet therapy was associated with a decreased incidence of acute respiratory distress syndrome/acute lung injury (odds ratio 0.50, 95% confidence interval 0.35–0.71) and reduced need of mechanical ventilation (odds ratio 0.62, 95% confidence interval 0.45–87). Incidence of acute kidney injury was similar between both groups (odds ratio 1.08, 95% confidence interval 0.73–1.59). *Conclusions*. The use of antiplatelet therapy before the diagnosis of severe sepsis or septic shock was not associated with decreased hospital mortality. Antiplatelet therapy was associated with a decreased incidence of acute lung injury/acute respiratory distress syndrome.

## 1. Introduction

Sepsis and its more severe forms (severe sepsis and septic shock) are frequent indications for Intensive Care Unit (ICU) admission [1] and commonly lead to multiple-organ dysfunction syndrome that is the main cause of death in critically ill patients [2].

There is strong evidence that sepsis, through the release of inflammatory mediators such as interleukin 1, 6 [3, 4], and tumor necrosis factor  *α*  [5], produces a dysfunctional activation of the hemostatic system [6, 7]. The resulting development of microvascular thrombosis is tightly related to associated organ failure and death [8]. Inflammation-driven activation of platelets plays a key role in this complex process [9] in which platelets are capable of recruiting and activating neutrophils, thereby, intensifying the inflammatory response.

Preclinical studies have shown beneficial effects of antiplatelets on the outcome of septic subjects [10–12]. Moreover, antiplatelet therapy has been associated with decreased incidence of systemic inflammatory response syndrome [13], decreased mortality in ICU patients [14–16] as well as with protective effects against the development of acute lung injury and acute respiratory distress syndrome (ALI/ARDS) [13, 17, 18].

In the present study, we aimed to compare the outcomes of critically ill patients admitted to a medical ICU who were on antiplatelet therapy before the development of severe sepsis or septic shock, versus critically ill patients who were not receiving antiplatelet therapies before the development of severe sepsis or septic shock, with appropriate adjustment for potentially confounding variables.

## 2. Patients and Methods

We performed a retrospective cohort study of severe sepsis and septic shock patients consecutively admitted to the medical ICU of an academic tertiary care center from January 2007 to December 2009.

Eligible patients were identified through an electronic search done with Mayo Clinic databases. Medical ICU discharge diagnoses of severe sepsis or septic shock based on International Classification of Diseases 9 (ICD9) were utilized to find these patients. ICD 9 codes used for performing the search were 038.9 (septicemia), 995.91 (sepsis), 995.92 (severe sepsis), and 785.52 (septic shock). Eligible patients' electronic medical records were manually reviewed, and only those who met the inclusion criteria were included in the final analysis. The exposure of interest was antiplatelet therapy at the time of ICU admission. Antiplatelet therapy was defined as documentation of use or administration of any acetyl salicylic acid-containing medication, clopidogrel, ticlopidine, or dipyridamole at the time of ICU admission in the medical record. Inclusion criteria were age ≥ 18 years, diagnosis of severe sepsis, or septic shock at the time of ICU admission and use of antiplatelet therapy before identification of severe sepsis or septic shock diagnosis. Exclusion criteria included mixed shock states as cardiogenic (including acute cardiogenic pulmonary edema and acute coronary syndrome), hemorrhagic, and obstructive shock (including cases of pulmonary embolism), readmission to ICU due to sepsis during the period of study, transfer from another hospital ICU, lack of research authorization for reviewing medical records, and comfort care status within the first 24 of ICU admission. Mayo Clinic Institutional Review Board approved the study protocol and waived the need for informed consent.

Demographic data, physiologic scores, and mortality-related outcomes were automatically retrieved from DataMart [19] that is a near-real-time electronic database developed and validated at Mayo Clinic which imports data from electronic medical records. Acute Physiology and Chronic Health Evaluation (APACHE) III score [20] was calculated one hour after ICU admission, due to the potential for cause-effect inversion with the traditional APACHE III score at 24 hours after ICU admission [18]. Information regarding comorbidities, type and source of infection, resuscitation variables, antibiotic treatment, laboratory values, organ failure scores, and use of any support therapy was obtained from the electronic medical records.

The main outcome measure was hospital mortality. Secondary outcomes were ICU mortality, hospital and ICU length of stay, development of ARDS-ALI, acute kidney injury (AKI), and need of mechanical ventilation and renal replacement therapy [21].

All patients underwent an institutional protocol for early goal directed therapy (EGDT) [22], with achievement of resuscitation goals at any point within the first six hours considered adequate goal-directed resuscitation. Antibiotic therapy was chosen and started concomitantly with resuscitation as guided by institutional protocols. Severe sepsis and septic shock were defined according to the American College of Chest Physicians/Society of Critical Care Medicine consensus conference criteria [23]. Severe sepsis and septic shock onset time was defined as suspicion of infection and fulfillment of two of four criteria for the systemic inflammatory response syndrome (temperature >38.3°C or <35.6°C, heart rate >90 beats/min, respiratory rate >20/min, or white blood cell [16, 21] count >12.0 × 10^3^ or <4.0 × 10^3^) plus any of these conditions: systolic blood pressure not higher than 90 mm Hg after a crystalloid-fluid challenge of 20 mL per kilogram of body weight, blood lactate concentration ≥4 mmol/L, or need of vasopressors [24]. 

ALI and ARDS were diagnosed based on the criteria outlined by the American-European consensus conference on ARDS [25]. AKI was defined according to the Acute Kidney Injury Network (AKIN) criteria [26]. The AKIN class was determined based on the worst of either creatinine criteria or urine output (UO) criteria (based on the hourly urine output). Baseline creatinine measurements were available for most patients. For those patients without baseline creatinine values, these were calculated by the formula: baseline creatinine = 0.74 − 0.2 (if female) + 0.08 (if black) + 0.003 × age (years) [27]. Patients were classified according to the maximum AKIN class reached during their ICU stay.

Continuous variables are presented as medians with 25%–75% interquartile range, while categorical data are summarized as frequencies with percentages. Predictor variables and the outcomes of interest were initially compared between patients who received antiplatelet therapy before the development of severe sepsis or septic shock and those that did not with univariate analyses. Differences in population distribution of continuous variables were assessed with the Wilcoxon rank sum test. Differences in proportions were tested using Chi-squared test or Fisher's exact test where appropriate. 

To reduce the risk of unequal distribution of important covariates between groups due to the lack of random assignment in this observational study, a propensity score analysis was performed. The propensity score, that is, the probability of receiving antiplatelet therapy, was calculated from a multiple logistic regression model based on the next variables that are associated with an increased prescription of antiplatelet therapy: age, smoking history, alcohol abuse, atrial fibrillation, hypertension, diabetes mellitus, coronary artery disease, cerebrovascular disease, peripheral artery disease, heart failure, and statin treatment. To further test the hypothesis that antiplatelet therapy was associated with decreased hospital mortality, the Cochran-Mantel-Haenszel estimate of the pooled odds ratio was determined after stratifying the antiplatelet therapy propensity scores into equally sized quintiles. This approach allowed full use of the data and also provided stratum-by-stratum estimates of the antiplatelet therapy odds ratio to better understand the association with hospital mortality. Importantly, the empiric distributions of propensity scores were inherently different between the antiplatelet-treated and nontreated patients. To address this issue more fully, a sensitivity analysis was performed matching antiplatelet-exposed patients to non-exposed patients based on propensity score. A 0.1 caliper of propensity was used when identifying each antiplatelet-exposed patient's non-exposed control(s). If no match could be identified for an antiplatelet-exposed patient, the patient was excluded from the analysis. Conditional logistic regression was used to estimate the antiplatelet treatment effect while conditioning on each matched set of case and control(s) (stratum) and adjusting for severity of disease using the APACHE III score. Odds ratio (OR) and 95% confidence intervals (CI) were calculated, and  *P*  values of <0.05 were considered statistically significant. All statistical analyses were performed using JMP statistical software base version 8.0 and SAS 9.1.4 (SAS Institute Inc, Cary, NC).

## 3. Results

**Figure 1:**
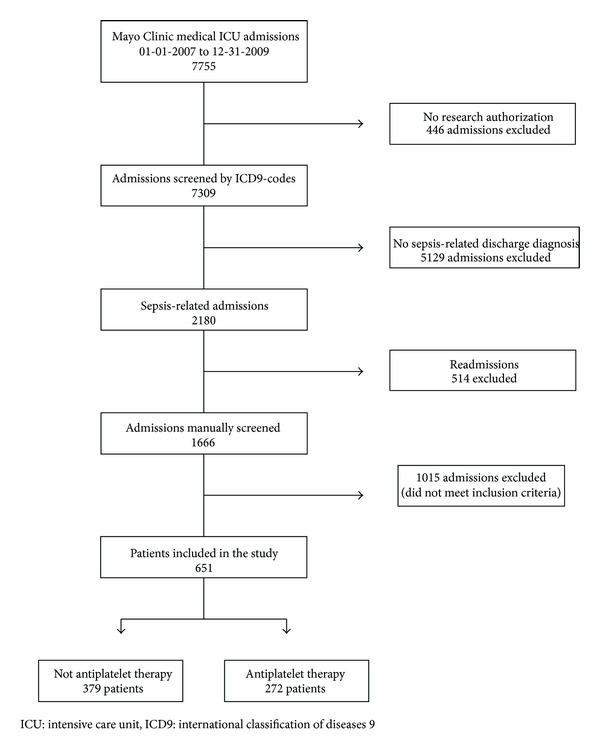
Study population.

Out of 7755 patients admitted to the medical ICU during the study period, 651 met the inclusion criteria and were included in the study (Figure 1: study population). Patients on antiplatelet therapy were older and had more comorbidities than patients who did not receive these medications (Table 1: Baseline demographic and clinical characteristics). The main antiplatelet drug used was aspirin as a single drug (88.6%) or in combination with clopidogrel (9.9%). Antiplatelet therapy was discontinued in 48 (17.6%) of the patients who received it before ICU admission.

Baseline physiologic and laboratory values between the two groups at the time of ICU admission are described in Table 2 (physiologic scores and laboratory values at the time of ICU admission). Of note, patients on antiplatelet had higher APACHE III scores, higher platelet counts, and lower prothrombin times. There were no significant differences in the resuscitation goals at 6 hours (Table 3: resuscitation and interventions assessment). 

Main outcomes are described in Table 4 (outcomes). In the univariate analyses, there was no statistical difference in hospital mortality, ICU mortality, hospital or ICU length of stay, AKI development, and need for renal replacement therapy between patients on antiplatelet therapy and those who did not receive antiplatelet therapy. The incidence of ARDS-ALI and need of invasive mechanical ventilation were significantly lower in the group that received antiplatelet therapy. After adjusting for propensity score using the Cochran-Mantel-Haenszel estimate of odds ratio weighted average over the 5 strata, antiplatelet therapy was associated with a reduction in hospital mortality, OR 0.65 (0.44 to 0.96),  *P* = 0.0320  (Table 5: propensity score analyses evaluating the association between pre-ICU admission antiplatelet therapy and hospital death after stratifying by quintile of antiplatelet propensity). A total of 180 patients on antiplatelet therapy were matched with 180 unexposed patients utilizing the propensity score and adjusting for severity of disease as predicted by APACHE III. After adjusting for these variables, antiplatelet therapy was not associated with a decreased risk of mortality, OR 0.73 (0.46–1.16),  *P* = 0.19.

## 4. Discussion

In the present study we found that chronic use of antiplatelet therapy before the diagnosis of severe sepsis or septic shock was not significantly associated with decreased hospital mortality in patients admitted to the medical ICU of a tertiary care center. Even though there was initially a trend for decreased mortality in the group who received antiplatelet therapy, this effect was no longer present after matching by propensity score and adjusting for the severity of disease.

Another important finding of our study was the unadjusted decreased trend for the development of ARDS/ALI in the group of subjects that received antiplatelet therapy and, as a consequence, less need of invasive mechanical ventilation in the group who received it. There was no association between the use of antiplatelet therapy and the development of AKI, which was very high in both groups. The high incidence of AKI was expected because, according to the AKIN criteria, a 0.3 mg/dL increase in baseline serum creatinine or an urine output less than 0.5 mL/Kg/h for more than 6 hours classified the patient as having stage 1 AKI, and, approximately a half of patients who developed AKI belong to this stage. Needs of renal replacement therapy were similar between both groups.

A potentially beneficial effect of antiplatelet therapy on septic patients can be explained by several mechanisms. The irreversible inhibition of platelet function caused by these drugs impedes their activation and surface expression of adhesion molecules like selectins and GPIIbIIIa receptors [28] which is a key step in the formation of microvascular thrombi [29]. Microvascular thrombosis causes ischemia and is thought to contribute to tissue injury and multiple organ dysfunction syndrome [30]. Moreover, platelet inactivation with antiplatelet therapies is believed to attenuate the secretion of inflammatory mediators [31, 32], depressing their interaction with immune cells [33] and thereby modulating the adverse effects associated with the inflammatory reactions [34]. 

Aspirin has shown some specific antiinflammatory beneficial effects in sepsis. It stimulates the synthesis of 15-epi-lipoxin A4, which in turn increases nitric oxide synthesis through endothelial nitric oxide synthase and inducible nitric oxide synthase [35]. Nitric oxide inhibits the interactions between leucocytes and endothelial cells, decreasing polymorph neutrophil recruitment [36]. Another putative effect of aspirin is the inhibition of the nuclear factor kappa-B [37], which plays an essential role in immune and inflammatory responses [38]. Due to the fact that most patients in our study cohort were on low-dose aspirin (98.5%), the beneficial effects of antiplatelet therapy in our cohort of severe sepsis and septic shock patients are probably related to this particular drug.

Lung is one of the most frequently injured organs in septic patients [39], and platelets are postulated to play a key role in the development of ARDS-ALI. Once they adhere to the vascular endothelium and activate their interaction with neutrophils [40] that produces endothelial and epithelial injury increasing vascular permeability [41] that leads to fluid and protein accumulation in the alveoli. We found a significant association between the use of antiplatelet therapy and a decreased incidence of ARDS-ALI. However, it should be mentioned that this hypothesis has been tested in previous studies, yielding conflicting results [13, 17, 18].

Sepsis is the main condition associated with the development of acute kidney injury in critically ill patients [42]. Its pathophysiology is complex and not fully understood. The role of platelets in the development of acute kidney injury has not been studied in humans, but preclinical research has found that their activation may be a prerequisite for the occurrence of kidney injury induced by endotoxin and that antiplatelets significantly restore some of the parameters of renal dysfunction produced by sepsis [43]. However in our study antiplatelet therapy was not associated with decreased incidence of AKI or use of renal replacement therapy.

Though antiplatelet agents are known to increase the risk of bleeding [44], patients who received antiplatelet therapy in this investigation did not require transfusion of blood products more frequently than patients who did not. Indeed, the antiplatelet cohort was transfused platelets less frequently than the nonantiplatelet exposed group. Moreover, the prothrombin time values were lower in those who were receiving antiplatelet therapy. These findings are consistent with the hypothesis that antiplatelets decrease the formation of microvascular thrombi and prevent the development of disseminated intravascular coagulation and the subsequent platelets and coagulation factors consumption. Although the present study was neither designed nor adequately powered to specifically address this potential association, we have previously reported such an association in a separate cohort of patients with septic shock [45].

Our study has the strength that all patients, since they arrived to the emergency department, were aggressively resuscitated according to the principles of EDGT and received antibiotics in an early stage of the their septic process which reassures that the subsequent development of shock and organ failure was not due to inadequate resuscitation. However, our study has also several limitations worth noting. The first major limitation is its retrospective design with the inherent concerns of all observational study designs, particularly unmeasured bias and confounding. To limit this concern, we performed adjusted analyses taking into account the study participant's antiplatelet propensity score. In addition, the observational nature of this study precluded assurances regarding the actual administration of the documented antiplatelet therapies. This concern is at least partially mitigated by the extended effect of antiplatelet therapies (e.g., 7 days or longer for the two most commonly utilized medications in this investigation—aspirin and clopidogrel). As a third potential limitation, it is also possible that the presence of antiplatelet therapy is a marker for more consistent health care maintenance, closer contact with the heath care system, and better control of the study of participant's baseline comorbidities. While a potential confounding issue, we believe that this concern is at least partially mitigated by the wide availability of health care resources in this study population and the significantly higher age and presence of comorbidities in patients who received antiplatelets. A fourth limitation relates to the loss of study participants in the propensity-matched analysis of hospital mortality. As a result of the matching procedures, we were only able to evaluate 180 pairs of antiplatelet-treated and -untreated participants. The resulting loss of statistical power may, in part, explain the discrepant results found using the Cochran Mantel Hansel test and the conditional logistic regression analysis for testing the association between antiplatelet therapy and hospital mortality. This concern is supported by the similar effect estimates, despite the loss of statistical significance. Finally, it should be mentioned that this study was performed in a single, academic, and tertiary care medical center whose patients are predominantly Caucasians. The resulting homogeneity of the study population may limit our ability to generalize these study's findings to more heterogeneous study populations and health care facilities. 

## 5. Conclusions

In a cohort of severe sepsis and septic shock patients treated according to the principles of Early Goal Directed Therapy, chronic antiplatelet therapy was not associated with decreased hospital or ICU mortality after adjusting for the propensity to receive antiplatelet therapy and severity of illness as calculated by APACHE III score. However, it is potentially protective against the development of acute lung injury/acute respiratory distress syndrome and reduces the needs of mechanical ventilation without increasing the requirements of blood product transfusions. The role of antiplatelets as an adjunctive therapy in the treatment of severe sepsis should be explored in prospective studies.

## Figures and Tables

**Table 1:** Baseline demographic and clinical characteristics.

	No antiplatelets *N* = 379	Antiplatelets *N* = 272	*P* value
Age, median (IQR)	65.8 (53.2 to 78.3)	75.2 (65.2 to 82.5)	<0.001
Female gender, *n* (%)	177 (46.7)	112 (41.2)	0.16
BMI, Kg/m², median (IQR)	27.4 (23 to 32.8)	29 (24.5 to 34.5)	0.015
Caucasian, *n* (%)	340 (89.6)	251 (92.3)	0.26
Admission source, *n* (%)			
Emergency department	205 (54.1)	162 (69.6)	0.17
Medicine wards	107 (28.2)	69 (25.4)	0.42
Outpatient clinic	55 (14.5)	28 (10.3)	0.11
Postoperative	10 (2.6)	7 (2.6)	0.96
Another in-hospital ICU	2 (0.53)	6 (2.2)	0.073
Comorbidities			
Smoking history, *n* (%)	88 (23.2)	70 (25.8)	0.46
Chronic alcohol use, *n* (%)	48 (12.7)	17 (6.3)	0.0058
Hypertension, *n* (%)	175 (46.2)	200 (73.5)	<0.001
Diabetes mellitus, *n* (%)	113 (29.8)	128 (47)	<0.001
Dyslipidemia, *n* (%)	101 (26.7)	125 (46)	<0.001
Coronary artery disease, *n* (%)	58 (15.3)	152 (55.9)	<0.001
Cerebrovascular disease, *n* (%)	37 (9.8)	60 (22)	<0.001
Peripheral artery disease, *n* (%)	34 (9)	63 (23.2)	<0.001
Heart failure, *n* (%)	17 (4.5)	38 (14)	<0.001
Atrial fibrillation, *n* (%)	62 (16.4)	66 (24.3)	0.012
Chronic obstructive pulmonary disease, *n* (%)	59 (15.6)	68 (25)	0.0027
Chronic kidney disease, *n* (%)	65 (17.2)	79 (29)	<0.001
Stage V chronic kidney disease, *n* (%)	30 (7.9)	30 (11)	0.18
Active neoplasia, *n* (%)	51 (13.5)	37 (13.6)	0.96
Chronic anticoagulation, *n* (%)	66 (17.4)	45 (16.5)	0.77
Statin therapy, *n* (%)	78 (20.6)	139 (51.1)	<0.001
Pharmacological immunosuppression, *n* (%)	65 (17.2)	38 (14)	0.27
Charlson score, median (IQR)	4 (1 to 6)	6 (5 to 8)	<0.001
Type of antiplatelet, *n* (%)			
Aspirin		241 (88.6)	
Aspirin and clopidogrel		27 (9.9)	
Clopidogrel only		4 (1.5)	
Aspirin dose, mg, median (IQR)		81 (81-81)	
Type of infection *n* (%)			
Community acquired	173 (45.6)	123 (45.2)	0.91
Health care related/nosocomial	206 (54.4)	149 (54.8)	0.91
Source of infection, *n* (%)			
Respiratory	139 (36.7)	82 (30.1)	0.083
Intraabdominal	73 (19.3)	43 (15.8)	0.26
Urinary	60 (15.8)	62 (22.8)	0.025
Skin and soft tissue	45 (11.9)	43 (15.8)	0.15
Line related	37 (9.8)	15 (5.5)	0.002
Unknown	17 (4.5)	18 (6.6)	0.23
Other	8 (2.1)	9 (3.3)	0.34
Positive cultures	261 (68.9)	182 (66.9)	0.6

IQR: interquartile range, *n*: number, %: percentage, BMI: body mass index, ICU: intensive care unit.

**Table 2:** Physiologic scores and laboratory values at the time of ICU admission.

	No antiplatelets *N* = 379	Antiplatelets *N* = 272	*P* value
APACHE III 1 hour, median (IQR)	55 (42–68)	57.5 (46–74.8)	0.025
APS 1 hour, median (IQR)	35 (24–49)	36 (25–49.8)	0.64
Predicted hospital death %, median (IQR)	9.2 (4.9–17.4)	10.4 (5.3–21.6)	0.089
SOFA score day 1, median (IQR)	7 (4–10)	6 (4–9)	0.054
ICU admission laboratory results			
Hemoglobin, g/dL, median (IQR)	10.9 (9.4–12.5)	11.1 (9.5–12.6)	0.46
Hematocrit %, median (IQR)	32 (27.9–37)	32 (28.3–36.9)	0.45
White blood cells, number ∗ 1000/dL, median (IQR)	12.3 (7.7–18.7)	14.3 (9.4–19.5)	0.017
Platelets, number ∗ 1000/dL, median (IQR)	191 (122–285)	212 (143–307)	0.012
Prothrombin time, sec, median (IQR)	12.8 (10.9–18)	11.9 (10.5–15)	0.01
International normalized ratio, median (IQR)	1.3 (1.1–1.9)	1.2 (1.1–1.6)	0.01
Bicarbonate, mmol/L, median (IQR)	22 (19–25)	22 (18–26)	0.86
Base excess, mmol/L, median (IQR)	4 (8–0)	4 (8–0)	0.76
Lactate, mmol/L, median (IQR)	2.3 (1.3–3.6)	2 (1.3–3.5)	0.56
Baseline creatinine, mg/dL (IQR)	0.9 (0.7–1.1)	1 (0.8–1.5)	<0.001
PaO_2_/FiO_2_ ratio, median (IQR)	256 (157–382)	278 (164–381)	0.57

APACHE: Acute Physiologic and Chronic Health Evaluation, IQR: interquartile range, APS: Acute Physiology Score, SOFA: Sequential Organ Failure Assessment, %: percentage, g/dL: grams/deciliter, sec: seconds, mmol/L: millimol/liter, mg/dL: milligrams/deciliter, PaO_2_/FiO_2_: relation arterial partial pressure of oxygen/fraction of inspired oxygen.

**Table 3:** Resuscitation and interventions assessment.

	No antiplatelets *N* = 379	Antiplatelets *N* = 272	*P* value
Time to first antibiotic, hours, median (IQR)*	0.25 (−2–1.3)	0.33 (−2.1–1.2)	0.82
Components of resuscitation at 6 hours			
MAP, mm Hg, median (IQR)	68 (62–76)	66 (59–76)	0.12
Urine output, mL/Kg/h, median (IQR)	0.54 (0.24–1.18)	0.46 (0.19–1)	0.25
Central venous pressure, median (IQR)			
Patients on MV	12 (9–16)	12 (7–16)	0.69
Patients without MV	8 (5–13)	8 (5–12)	0.92
ScVO_2_, %, median, (IQR)	74 (67–78)	71 (64–76)	0.02
Transfusion therapy during ICU hospitalization			
Blood product transfusion, number of patients (%)	187 (49.3)	127 (46.7)	0.50
Red blood cells transfusion, number of patients (%)	161 (42.5)	114 (41.9)	0.89
Red blood cells units transfused, number, median (IQR)	2 (2–4)	2 (2-3)	0.27
Platelets transfusion, number of patients (%)	41 (10.8)	14 (5.1)	0.01
Platelet units transfused, number, median (IQR)	2 (1–5.5)	2 (1–3.3)	0.58
Fresh frozen plasma transfusion, number of patients (%)	64 (16.9)	38 (14)	0.31
Fresh frozen plasma units transfused, number, median (IQR)	2 (2–6)	2.5 (2–4)	0.14
Cryoprecipitates transfusion, number of patients (%)	10 (2.6)	8 (2.9)	0.82
Cryoprecipitates units transfused, number, median (IQR)	4 (2–6)	2 (2–3.5)	0.14
Adjuvant therapy			
Stress dose steroids, *n* (%)	143 (37.3)	86 (31.6)	0.11
Drotrecogin alfa activated, *n* (%)	7 (1.9)	0	0.046
Vasopressors, *n* (%)	231 (61)	149 (54.8)	0.12
Length of vasopressors, hours, median (IQR)	33 (12–75)	28 (13–54.5)	0.29

IQR: interquartile range, MAP: mean arterial pressure, mm Hg: millimeters of mercury, mL/Kg/h: milliliters/kilogram/hour, ScVO_2_: central venous oxygen saturation, %: percentage, *n*: number, ICU: intensive care unit.

*Time from first antibiotic to severe sepsis or septic shock diagnosis.

**Table 4:** Outcomes.

	No antiplatelets *N* = 379	Antiplatelets *N* = 272	O.R. (95% CI)	*P* value
Hospital death *n* (%)	98 (25.9)	57 (21)	0.76 (0.52–1.10)	0.15
ICU death, *n* (%)	46 (12.1)	26 (9.6)	0.77 (0.46–1.27)	0.3
Hospital length of stay days, median (IQR)	9.3 (5.5–16.9)	8.9 (5.6–15.7)		0.86
ICU length of stay days, median (IQR)	2.5 (1.3–5.8)	2.3 (1.3–4.7)		0.31
ARDS/ALI, *n* (%)	132 (34.8)	57 (21)	0.50 (0.35–0.71)	<0.001
Invasive mechanical ventilation, *n* (%)	158 (41.7)	84 (30.9)	0.62 (0.45–0.87)	0.005
Length of invasive mechanical ventilation days, median (IQR)	3.3 (1.3–8)	3.5 (1.8–7)		0.99
Noninvasive mechanical ventilation, *n* (%)	68 (17.9)	50 (18.4)	1.03 (0.64–1.54)	0.89
Length of noninvasive mechanical ventilation days, median (IQR)	0.64 (0.2–1.8)	1 (0.4–2)		0.18
Acute kidney injury, *n* (%)	299 (78.9)	218 (80.2)	1.08 (0.73–1.59)	0.70
Stage of acute kidney injury				
Stage 1, *n* (%)	126 (33.3)	102 (37.5)		NS
Stage 2, *n* (%)	63 (16.6)	44 (16.2)		NS
Stage 3, *n* (%)	110 (29)	72 (26.5)		NS
Renal replacement therapy, *n* (%)	85 (22.4)	53 (19.5)	0.84 (0.57–1.23)	0.37
Length of renal replacement therapy days, median (IQR)	6 (3–11)	4 (2–8)		0.22

*n*: number, %: percentage, IQR: interquartile range, ARDS/ALI: acute respiratory distress syndrome/acute lung injury.

**Table 5:** Propensity score analysis evaluating the association between pre-ICU admission antiplatelet therapy and hospital death after stratifying by quintile of antiplatelet propensity.

Strata	Propensity score range	Odds ratio (95% CI)	*P* value
1 (*n* = 167)	0–0.20	0.61 (0.17–2.23)	0.45
2 (*n* = 199)	0.21–0.40	0.66 (0.34–1.27)	0.21
3 (*n* = 110)	0.41–0.60	1.01 (0.47–2.16)	0.97
4 (*n* = 115)	0.61–0.80	0.35 (0.14–0.87)	0.02
5 (*n* = 60)	0.81–1	0.52 (0.12–2.21)	0.37

Overall (*n* = 651)		0.65 (0.44–0.96)	0.03

*n*: number.
